# Unraveling the genetic association between obesity-related anthropometric indices and foot deformities in the European population: A two-sample Mendelian randomization study

**DOI:** 10.1097/MD.0000000000047087

**Published:** 2026-01-09

**Authors:** Zhenyu Cai, Le Chang, Rongdong Zeng

**Affiliations:** a Department of Orthopedics, Quanzhou First Hospital Affiliated to Fujian Medical University, Quanzhou, Fujian Province, China; b Department of Clinical Medicine, Quanzhou Medical College, Quanzhou, Fujian Province, China.

**Keywords:** BMI, flat foot, foot deformities, hallux valgus, Mendelian randomization, obesity

## Abstract

Obesity and foot deformities pose substantial public health burdens globally. However, their causal relationship remains uncertain due to confounding and reverse causation in observational studies. This study aimed to investigate causal associations between obesity-related anthropometric indices and foot deformities using Mendelian randomization (MR) analysis. This two-sample MR study utilized genetic instruments from publicly available genome-wide association studies databases. We extracted single nucleotide polymorphisms (SNPs) as instrumental variables for obesity (6 SNPs), body mass index (372 SNPs), waist circumference (257 SNPs), and hip circumference (313 SNPs). The outcomes included flat foot and hallux valgus from the FinnGen Biobank. Inverse variance weighted method was used as the primary analysis, supplemented by MR-Egger, weighted median, simple mode, and weighted mode. Sensitivity analyses included heterogeneity tests and pleiotropy assessments. Genetically predicted obesity showed significant causal effects on flat foot (odds ratio [OR]: 1.516, 95% confidence interval [CI]: 1.166–1.970, *P* = .002) and hallux valgus (OR: 1.179, 95% CI: 1.041–1.336, *P* = .010). Similar associations were observed for body mass index (flat foot: OR: 1.798, 95% CI: 1.491–2.168, *P* < .001; hallux valgus: OR: 1.193, 95% CI: 1.075–1.324, *P* = .001), waist circumference (flat foot: OR: 1.542, 95% CI: 1.210–1.966, *P* < .001; hallux valgus: OR: 1.270, 95% CI: 1.108–1.456, *P* = .001), and hip circumference (flat foot: OR: 1.380, 95% CI: 1.134–1.680, *P* = .001; hallux valgus: OR: 1.154, 95% CI: 1.009–1.319, *P* = .036). This genetic study provides strong evidence supporting causal relationships between obesity-related anthropometric indices and foot deformities. These findings emphasize the importance of weight management in preventing foot deformities and suggest potential therapeutic targets for intervention strategies.

## 1. Introduction

Obesity has emerged as a critical global public health challenge. According to the latest systematic analysis published in The Lancet, from 1990 to 2022, the prevalence of obesity has increased substantially worldwide, with particularly concerning trends in both developed and developing countries.^[1]^ According to a comprehensive analysis published in Metabolism in 2022, obesity prevalence in Europe increased substantially from 8.4% in 1980 to 20% in 2019, with considerable regional variation.^[2]^ Southern European countries demonstrate particularly high prevalence rates, with projections suggesting that adult obesity in some of the most affected countries may approach 40% by 2035.^[3]^

Foot deformities, particularly flatfoot and hallux valgus, represent significant musculoskeletal conditions affecting global population health. A recent systematic review and meta-analysis revealed that the global prevalence of hallux valgus is approximately 19% (95% confidence interval [CI], 13–25%), with significant regional variations.^[4]^ Flatfoot, characterized by reduced medial longitudinal arch height, affects approximately 13% to 27% of adults globally, with higher prevalence rates observed in specific populations.^[5]^ A comprehensive systematic review and meta-analysis published in 2023 reported that the pooled prevalence of hallux valgus in Europe was 18.35% based on 18 studies involving 114,526 participants from 7 European countries,^[4]^ while flatfoot prevalence in European adult populations ranges from approximately 19% to 27%, with a representative study in Spain reporting a prevalence of 26.62% among adults aged 40 years and above.^[6]^ These conditions significantly impact mobility and quality of life, with studies indicating associations with chronic pain, functional limitations, and increased fall risk.^[7]^

Recent studies have demonstrated complex associations between obesity and foot deformities. Systematic reviews and meta-analyses have shown that increased body mass index (BMI) significantly alters foot morphology, with obese individuals demonstrating a 2.3-fold increased risk of developing flat foot (95% CI: 1.7–3.1) and associated changes in plantar pressure distribution.^[8]^ However, longitudinal cohort studies have yielded conflicting results, particularly regarding hallux valgus: while some researchers report a positive correlation between BMI and hallux valgus angle progression, others found that foot morphology alterations in overweight children showed more complex patterns, with variations in arch height not consistently correlating with BMI.^[9]^ Given these contradictory findings, there is a critical need for well-designed prospective studies to elucidate the temporal relationship between obesity and foot deformities, as recent evidence suggests that the association may be modulated by age, gender, and genetic predisposition.^[10]^

Contemporary genetic epidemiology has been revolutionized by high-throughput genomic screening technologies, particularly through the implementation of genome-wide association studies (GWAS), which have unveiled unprecedented insights into the genetic architecture of complex traits.^[11]^ The integration of sophisticated statistical frameworks, notably Mendelian randomization (MR), has emerged as a pivotal methodological advancement in causal inference. This innovative approach leverages genetic variants as natural experiments, exploiting the stochastic distribution of alleles during gametogenesis to establish causal relationships between phenotypic traits.^[12]^ The methodology employs carefully selected single nucleotide polymorphisms (SNPs) as instrumental variables (IVs), capitalizing on their unique biological properties (including temporal precedence to phenotype manifestation and robustness against environmental perturbations). Recent methodological refinements have introduced advanced analytical frameworks, including pleiotropy-robust methods and multivariable approaches, substantially enhancing the reliability of causal estimates in complex trait associations.^[13]^ In the context of investigating the relationship between adiposity and podiatric structural anomalies, this genetically-informed approach offers unprecedented opportunities to delineate causal pathways while circumventing traditional epidemiological constraints.

Based on genome-wide association study databases, this investigation employed a two-sample MR analysis approach with genetic IVs related to obesity to explore the causal relationship with foot deformities, providing genetic epidemiological evidence for elucidating the pathogenic mechanisms.

## 2. Methods

### 2.1. Study design

**Figure 1.**
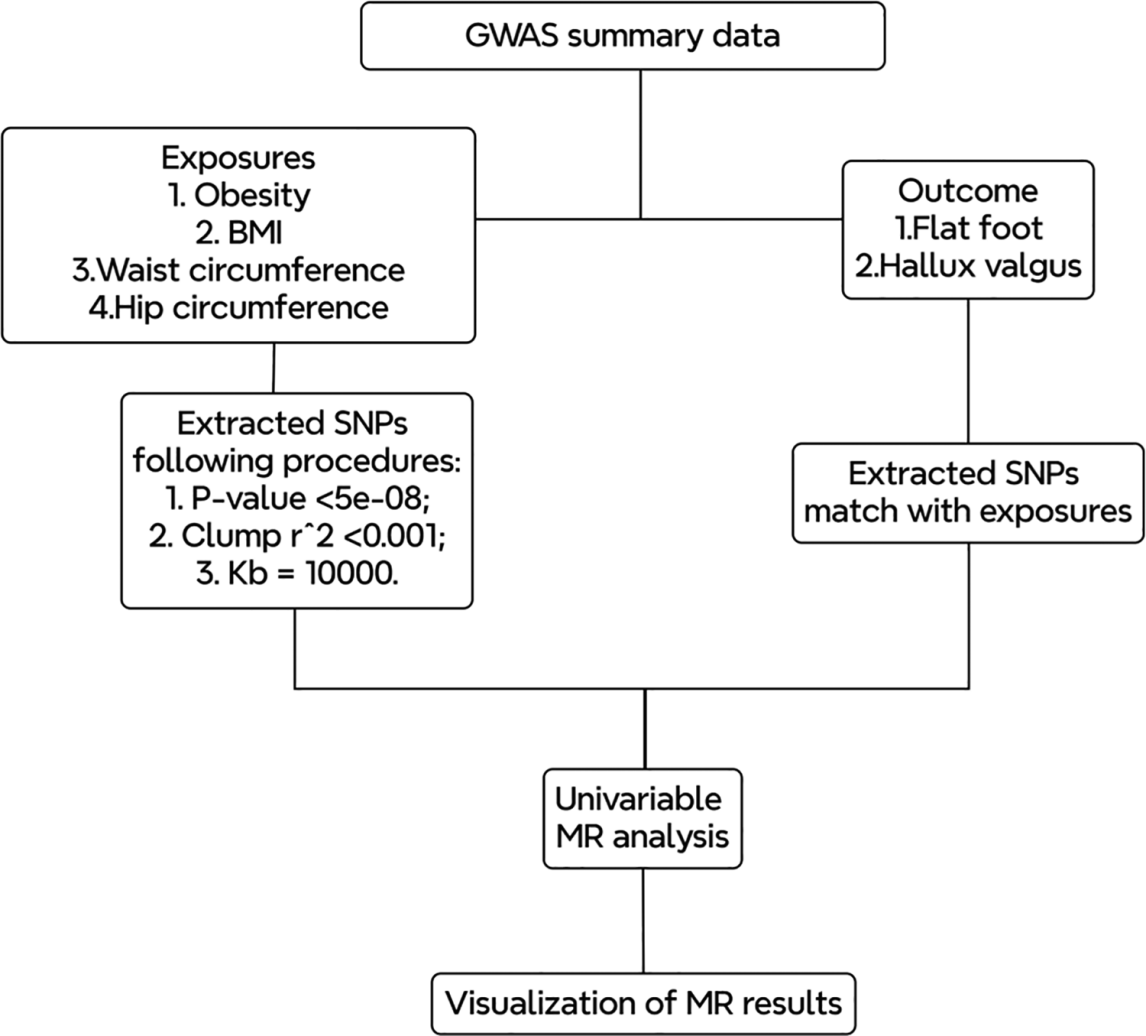
The MR analysis workflow. GWAS = genome-wide association studies, MR = Mendelian randomization, SNPs = single nucleotide polymorphisms.

In order to investigate the causal relationship between obesity-related anthropometric indices (including obesity, BMI, waist circumference, and hip circumference) and foot deformities (flat foot and hallux valgus), we utilized SNPs as IVs from the publicly accessible GWAS database from Gwas Catalog,^[14]^ Integrative Epidemiology Unit OpenGWAS project,^[15]^ and Finn biobank,^[16]^ thereby obviating the need for additional ethical approvals. Our analysis used two-sample MR to assess the potential causal relationships between obesity-related anthropometric indices and foot deformities. The entire MR analysis workflow is shown in Figure 1.

### 2.2. Data sources

The data sources of exposures and outcomes in the present study were obtained from different databases in order to avoid bias caused by the overlap samples. The exposures data of obesity was selected in GWAS Catalog project with the sample sizes of 408,792. GWAS summary for BMI, waist circumference and hip circumference were obtained from the Integrative Epidemiology Unit openGWAS project with sample sizes of 461,460, 407,661, and 407,661. GWAS summary data for flat foot and hallux valgus were obtained from the Finn Biobank database with sample sizes of 291,215 and 305,217. Table 1 provides details of the data sources used and the demographic profiles. The GWAS summary data used on this study was previously published so that additional ethical approval or consent to participate was not required.

**Table 1:** Data sources used in this study, overview of genome-wide association studies used in the analyses for the exposures/outcomes.

Exposures and outcomes	Year	Sample size	Ancestry	Consortia	GWAS ID	*F* statistics	*R*
Obesity	2018	408,792	European	Gwas Catalog	GCST90435786	50.510	0.074%
BMI	2018	461,460	European	IEU	ukb-b-19953	64.761	5.220%
Waist circumference	2021	407,661	European	IEU	ebi-a-GCST90014020	43.849	2.764%
Hip circumference	2021	407,661	European	IEU	ebi-a-GCST90014021	95.173	7.308%
Flat foot	2024	291,215	European	Finn biobank	finngen_R11_M13_FLATFOOT		
Hallux valgus	2024	305,217	European	Finn biobank	finngen_R11_M13_HALLUXVALGUS		

BMI = body mass index, IEU = Integrative Epidemiology Unit.

### 2.3. Genetic instruments selection

There are 3 core assumptions that need to be met for the present MR study^[17]^: IVs should be closely associated with exposure factors; the genome-wide SNPs are not associated with confounding factors between exposures and outcomes; and there are no any other causal biological mechanism between SNPs and outcomes other than influenced by exposures. SNPs from the European ancestry-based 1000 Genomes Project were regarded as IVs at genomic level of *P* < 5 × 10^‐8^, and linkage diseqilibrium *r*^2^ < 0.001 and physical distance between them was within 10,000 kb.^[18]^ Furthermore, we utilized *F*-statistics to assess the strength of the IVs, which could avoid the weak instrumental bias. IVs were considered to be strongly correlated with exposures while *F* statistics > 10. *F* = β^2^_exposure_/SE^2^_exposure_. SNPs with *F* < 10 will be excluded.^[19]^ To ensure the validity of instrumental variables and minimize potential confounding, we utilized the PhenoScanner V2 database (http://www.phenoscanner.medschl.cam.ac.uk/) to examine whether the selected SNPs were associated with potential confounders at genome-wide significance level. The PhenoScanner analysis revealed no significant associations between the selected SNPs.

### 2.4. Statistical analyses

MR analysis was performed to assess the relationship between obesity-related anthropometric indices and foot deformities. The inverse variance weighting Mendelian randomization method as the main method to estimate the causal relationship between exposure and outcome; MR-Egger, weighted median, simple mode and weighted mode were employed as the supplementary methods.^[20]^ The results were reported as odds ratios (ORs) with 95% CIs because of the type of variables in this MR analysis was binary categorical variable. We considered the significant causal relationship between exposures and outcomes while *P* values of inverse variance weighted (IVW) method below 0.05 and the supplementary methods were consistent with IVW. All statistical analyses were performed using the Two Sample MR (version 0.6.4; MRC Integrative Epidemiology Unit at the University of Bristol, Bristol, UK) package in R software version 4.3.2.

### 2.5. Sensitivity analysis

In the sensitivity analysis, heterogeneity test and pleiotropy test were employed to verify the stability and reliability. The Cochrane *Q* test was utilized to test for heterogeneity of IVs and *P* value > .05 reflect no heterogeneity. A random-effect model would be adopted in the subsequent analyses when heterogeneity existed, otherwise, a fixed-effect model would be adopted.^[21]^ The horizontal pleiotropy test was utilized to evaluate the presence of horizontal pleiotropy in the current study. The presence of horizontal pleiotropy can be considered as the occurrence of outcomes regardless of the exposures involved. To assess pleiotropy, we utilized the intercept term of MR-Egger regression because of *P* value above .05 indicates no pleiotropic effect of the IVs. Moreover, a significant horizontal pleiotropic effect of individual SNPs inducing bias was evaluated with “leave-one-out” sensitivity test.^[11]^

## 3. Results

### 3.1. The extracted SNPs for MR analyses

In the MR analysis, obesity-related anthropometric indices were used as exposures, and the IVs extracted from the relevant GWAS summary data were as follows: 6 SNPs as IVs for obesity, 372 SNPs for BMI, 257 SNPs related to waist circumference, 313 SNPs for hip circumference. *F*-statistics of all SNPs > 10 indicate that there are no weak IVs.

### 3.2. Causal relationship of obesity-related anthropometric indices and flat foot

**Figure 2.**
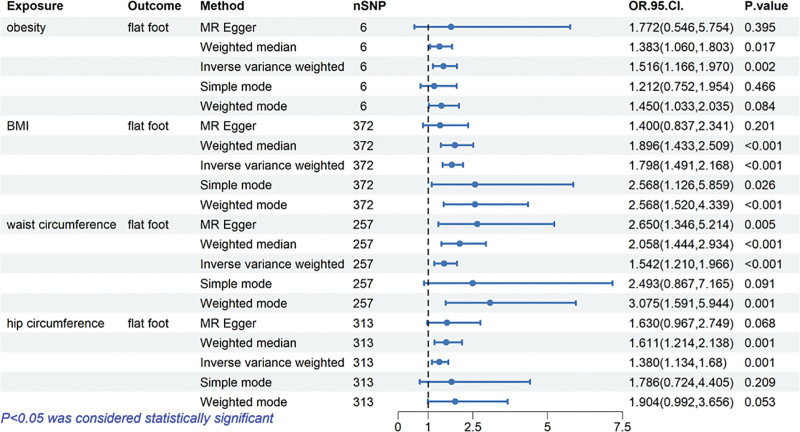
Forest plots of causal estimates in 2 sample Mendelian analysis of associations of genetic liability to obesity-related anthropometric indices with risk of flat foot.

**Figure 3.**
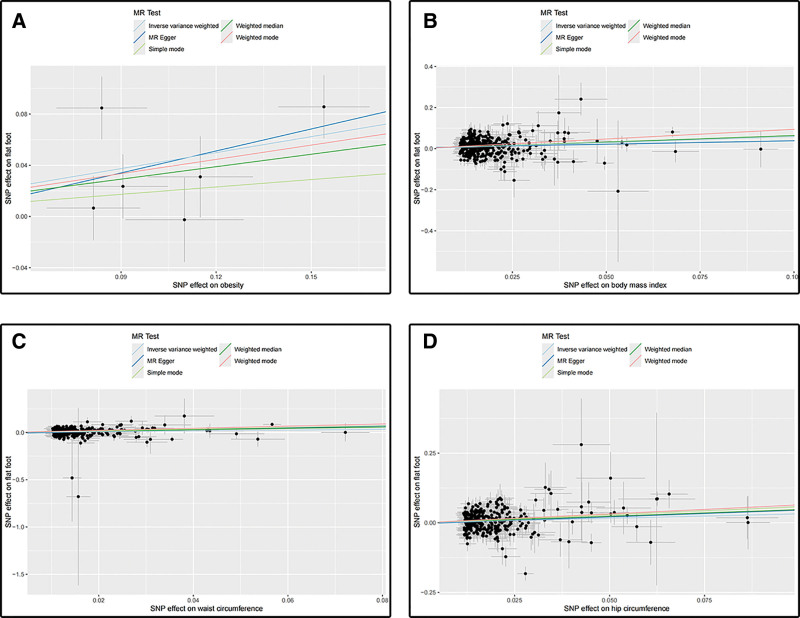
(A) Scatter plot for analysis of obesity and flat foot; (B) scatter plot for analysis of BMI and flat foot; (C) scatter plot for analysis of waist circumference and flat foot; (D) scatter plot for analysis of hip circumference and flat foot. BMI = body mass index.

The MR results for the causal effects of obesity-related anthropometric indices on flat foot are listed in Figures 2 and 3. The results obtained by the IVW method reflected that the causal relationship between obesity, BMI, waist circumference, hip circumference on flat foot. With the 1-standard deviation increase in obesity, the ORs of flat foot were 1.516 (95% CI: 1.166–1.970, *P* = .002) in the IVW method. In addition to BMI was also associated with flat foot (OR: 1.798, 95% CI: 1.491–2.168, *P* < .001). Genetically predicted waist circumference (OR: 1.542, 95% CI: 1.210–1.966, *P* < .001) and hip circumference (OR: 1.380, 95% CI: 1.134–1.680, *P* = .001) were also associated with the risk of flat foot. The “leave-one-out” analysis plots, funnel plots, and forest plots between obesity-related anthropometric indices on flat foot are presented in Figures S1–S12, Supplemental Digital Content, https://links.lww.com/MD/R103.

### 3.3. Causal relationship of obesity-related anthropometric indices and hallux valgus

**Figure 4.**
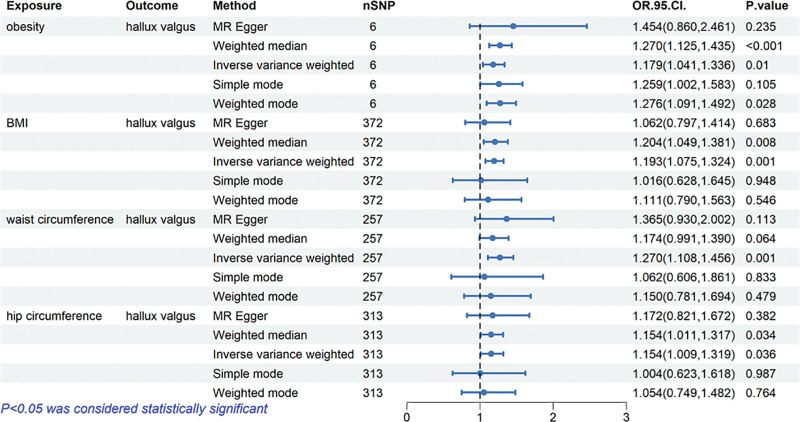
Forest plots of causal estimates in 2 sample Mendelian analysis of associations of genetic liability to obesity-related anthropometric indices with risk of hallux valgus.

**Figure 5.**
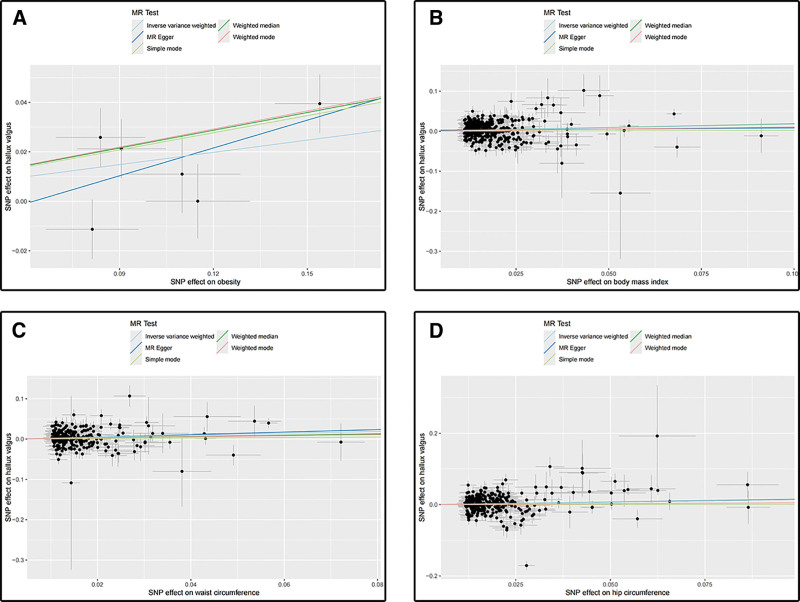
(A) Scatter plot for analysis of obesity and hallux valgus; (B) scatter plot for analysis of BMI and hallux valgus; (C) scatter plot for analysis of waist circumference and hallux valgus; (D) scatter plot for analysis of hip circumference and hallux valgus. BMI = body mass index.

In order to examine the associations between obesity-related anthropometric indices and hallux valgus, a two-sample MR analysis was conducted in the present study. The results of the MR analysis, depicted in Figures 4 and 5, confirmed the presence of positive causal effects between obesity-related anthropometric indices with hallux valgus. Specifically, using the IVW method, we found that obesity had a significant causal effect on hallux valgus (OR = 1.179; 95% CI: 1.041–1.336; *P* = .010), as well as BMI (OR = 1.193, 95% CI: 1.075–1.324; *P* = .001). The results showed that waist circumference and hip circumference were positively associated with the hallux valgus with statistical significance (OR_waist circumference_ = 1.270, 95% CI: 1.108–1.456; *P* = .001; OR_hip circumference_ = 1.154, 95% CI: 1.009–1.319; *P* = .036). These findings provide evidence for the positive causal relationship among obesity, BMI, waist circumference, hip circumference, and hallux valgus. Supplementary analyses including “leave-one-out” analysis plots, funnel plots, and forest plots examining the associations between obesity-related anthropometric indices with hallux valgus are presented in Figures S13–S24, Supplemental Digital Content, https://links.lww.com/MD/R103.

### 3.4. Sensitivity analysis

In Tables 2 and 3, the Cochran *Q* test for IVW method showed that obesity-related anthropometric indices had heterogeneity on foot deformities, thus all MR analysis was underwent on random-effect model. In the MR-Egger regression analysis to assess the pleiotropy (Tables 2 and 3), no evidence of pleiotropy was observed in the associations between exposures, and outcomes (all *P* > .05).

**Table 2:** MR sensitivity analyses of between exposures and flat foot.

Exposures or mediator	Outcome	Heterogeneity tests		Directional horizontal pleiotropy test	
		Methods	Cochran *Q* (*P*)	MR-Egger intercept	Pleiotropy *P*-value
Obesity	Flat foot	MR-Egger	8.677 (.070)	-0.017	.801
		Inverse variance weighted	8.833 (.116)		
BMI		MR-Egger	517.950 (<.001)	0.005	.306
		Inverse variance weighted	519.420 (<.001)		
Waist circumference		MR-Egger	334.191 (<.001)	-0.009	.095
		Inverse variance weighted	337.881 (<.001)		
Hip circumference		MR-Egger	438.654 (<.001)	-0.003	.501
		Inverse variance weighted	439.296 (<.001)		

BMI = body mass index.

**Table 3:** MR sensitivity analyses of between exposures and hallux valgus.

Exposures or mediator	Outcome	Heterogeneity tests		Directional horizontal pleiotropy test	
		Methods	Cochran *Q* (*P*)	MR-Egger intercept	Pleiotropy *P*-value
Obesity	Hallux valgus	MR-Egger	7.526 (.111)	‐0.023	.465
		Inverse variance weighted	8.752 (.119)		
BMI		MR-Egger	704.030 (<.001)	0.002	.391
		Inverse variance weighted	705.434 (<.001)		
Waist circumference		MR-Egger	463.032 (<.001)	‐0.001	.693
		Inverse variance weighted	463.318 (<.001)		
Hip circumference		MR-Egger	888.317 (<.001)	‐0.0003	.926
		Inverse variance weighted	888.342 (<.001)		

BMI = body mass index.

## 4. Discussion

Obesity is an escalating public health issue and is widely acknowledged as a significant risk factor for various chronic diseases. From a physiological perspective, obesity exhibits strong associations with multiple health complications such as cardiovascular disease, diabetes, hypertension, arthritis, and specific types of cancer.^[22–25]^ These diseases not only diminish individuals’ quality of life but also substantially augment medical expenses. Obesity subjects the body to heightened force and stress levels, particularly on the bones and joints of the lower extremities. For instance, the knee and hip joints are frequently affected areas. Excessive weight expedites joint cartilage deterioration while elevating the likelihood of developing arthritis, especially osteoarthritis.^[26]^

Based on the most recent scientific literature, a comprehensive systematic review and meta-analysis had demonstrated a substantial correlation between childhood obesity and the development of flat foot. The findings indicated that obese children have a 2.66-fold higher likelihood of developing flat foot compared to their normal-weight counterparts (95% CI: 1.76–4.02, *P* < .001).^[27]^ Recent cross-sectional study had corroborated these findings, showing that being overweight or obese is significantly associated with the development of flat foot in school-aged children (adjusted OR = 3.12, 95% CI: 2.31–4.18). Additionally, the study suggested that physical activity levels may moderate this association, with obese children who engage in less physical activity exhibiting a higher incidence of flat foot.^[28]^ In the adult population, a multicenter study involving 2445 participants revealed that for every 5-unit increment in BMI, the risk of developing flat foot increases by 51% (adjusted hazard ratio = 1.51, 95% CI: 1.24–1.84).^[29]^ The study also noted that the plantar pressure distribution in obese individuals is significantly altered, with increased weight-bearing in the midfoot region, which may be a critical mechanism underlying the development of flat foot.

Regarding the relationship between obesity and hallux valgus, a recently published systematic review and meta-analysis encompassing 32 studies with a total of 73,994 participants found an overall prevalence of hallux valgus at 23.0%. When examining risk factors, research has identified gender disparities in the association between obesity and hallux valgus. Specifically, among females, individuals with a BMI ≥ 30 exhibit a reduced risk of hallux valgus compared to those with a normal weight (relative risk = 0.74, 95% CI: 0.88–0.94).^[30]^ However, a subsequent prospective cohort study presented contrasting findings. This study monitored 1856 participants over a period of 5 years and determined that, after controlling for age, sex, and other confounding variables, each 5-unit increment in baseline BMI was associated with a 28% increase in the risk of hallux valgus (hazard ratio = 1.28, 95% CI: 1.12–1.47). The researchers hypothesize that these discrepancies could be attributed to variations in study design, follow-up duration, and participant demographics.^[31]^

The potential mechanisms of obesity leading to flat foot and hallux valgus can be explained from biomechanical and pathophysiological perspectives: excessive body weight causes abnormal mechanical loads on the feet, and this continuous pressure leads to alterations in the arch structure.^[32]^ Specifically, excess weight increases plantar pressure, particularly in the metatarsal heads and midfoot regions, which becomes especially pronounced during the terminal stance phase of gait.^[33]^ Chronic pressure loading results in prolonged stretching of foot ligaments, particularly the plantar fascia and posterior tibial tendon, ultimately leading to arch collapse and the development of flat foot.^[34]^ Simultaneously, as body weight increases, the foot compensatorily increases its contact area to distribute pressure, further exacerbating arch collapse. Regarding hallux valgus development, excessive weight increases pressure on the first metatarsophalangeal joint, leading to laxity of the joint capsule and medial collateral ligament, while creating an imbalance between abductor and adductor muscles, promoting gradual lateral deviation of the hallux.^[35]^ Furthermore, the presence of flat feet alters the biomechanical axis of the foot, further aggravating abnormal stress distribution in the first metatarsophalangeal joint, creating a vicious cycle that ultimately accelerates the progression of hallux valgus.^[36]^

In this study, our findings provide robust genetic evidence supporting significant causal associations between obesity-related indices and these foot conditions. Our analysis revealed compelling evidence that all investigated obesity-related indices, including obesity, BMI, waist circumference, and hip circumference, demonstrated significant causal effects on both flat foot and hallux valgus. Notably, for flat foot, BMI showed the strongest causal effect (OR = 1.798, 95% CI: 1.491–2.168), followed by obesity (OR = 1.516, 95% CI: 1.166–1.970) and waist circumference (OR = 1.542, 95% CI: 1.210–1.966), while hip circumference demonstrated a relatively weaker effect (OR = 1.380, 95% CI: 1.134–1.680). Regarding hallux valgus, waist circumference exhibited the strongest association (OR = 1.270, 95% CI: 1.108–1.456), while BMI and obesity showed similar effect sizes (OR = 1.193 and 1.179, respectively). These findings have significant clinical implications. They emphasize the importance of weight management in foot health and suggest that different obesity-related indices may have varying impacts on foot deformities. This understanding could inform more targeted prevention strategies and intervention approaches. The differential effects of various anthropometric indices also highlight the need for comprehensive assessment in clinical practice.

The methodological strengths of our study are noteworthy. First, the IVs datasets were obtained from different databases to mitigate the bias caused by sample overlap, and *F*-statistics exceeding 10 for all SNPs, ensuring robust statistical power.^[37]^ Second, multiple sensitivity analyses confirmed the robustness of our findings. Third, MR-Egger regression analysis revealed no evidence of pleiotropy (all *P* > .05), enhancing the reliability of our causal inference. Although heterogeneity was present, we appropriately addressed this through random-effects modeling.

However, several limitations should be acknowledged. While MR analysis effectively controls for confounding, unmeasured confounders may still exist. Additionally, as the genetic instruments were primarily derived from European populations, the generalizability of our findings to other ethnic groups requires further investigation. Furthermore, our study cannot fully elucidate the biological mechanisms underlying the observed associations. While our study focuses on direct causal effects through biomechanical pathways, obesity may also have indirect effects through lifestyle factors. Future multivariable MR studies could disentangle these pathways, although our sensitivity analyses suggest direct effects predominate.

Future research directions should focus on exploring the molecular mechanisms linking obesity to foot deformities, validating these findings in diverse populations, and conducting prospective intervention studies to evaluate the effectiveness of weight management in preventing foot deformities. From a public health perspective, our findings underscore the importance of implementing obesity prevention strategies as a means of reducing the risk of foot deformities.

## 5. Conclusion

This groundbreaking genetic study provides the first conclusive evidence that obesity is a significant cause of foot deformities, substantially influencing the development of both flat foot and hallux valgus. These findings establishing solid scientific evidence for the relationship between obesity and foot health. The results emphasize the critical role of weight management in preventing foot deformities, offering new directions for clinical intervention strategies.

Supplemental digital content “supplement datar” is available for this article (https://links.lww.com/MD/R102).

## Acknowledgments

We want to acknowledge the participants and investigators of the GWAS Catalog project, IEU OpenGWAS progect and FinnGen study.

## Author contributions

**Conceptualization:** Zhenyu Cai.

**Data curation:** Zhenyu Cai, Le Chang, Rongdong Zeng.

**Formal analysis:** Zhenyu Cai, Le Chang, Rongdong Zeng.

**Investigation:** Le Chang, Rongdong Zeng.

**Methodology:** Zhenyu Cai, Rongdong Zeng.

**Project administration:** Zhenyu Cai, Le Chang, Rongdong Zeng.

**Resources:** Le Chang, Rongdong Zeng.

**Software:** Le Chang, Rongdong Zeng.

**Validation:** Rongdong Zeng.

**Visualization:** Rongdong Zeng.

## Supplementary Material




