# Supplementary material for: Unraveling the genetic association between obesity-related anthropometric indices and foot deformities in the European population: A two-sample Mendelian randomization study
Source: Medicine (Baltimore). 2026 Jan 9;105(2):e47087. doi: 10.1097/MD.0000000000047087 (PMC12794961; doi:10.1097/MD.0000000000047087)
Supplement: Supplementary file 1 [file medi-105-e47087-s001.pdf]

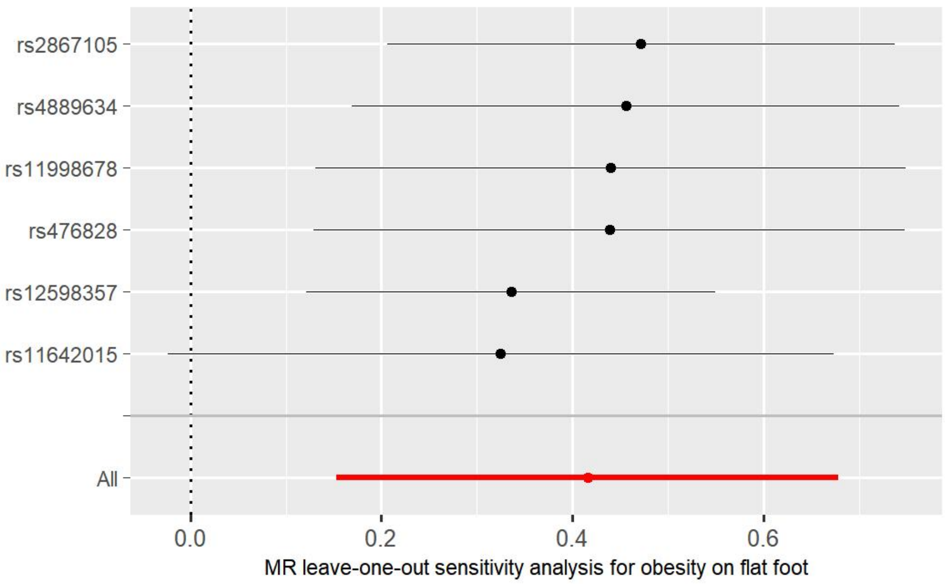
rs2867105
rs4889634
rs11998678
rs476828
rs12598357
rs11642015
All
0.0
0.2
0.4
0.6
MR leave-one-out sensitivity analysis for obesity on flat foot

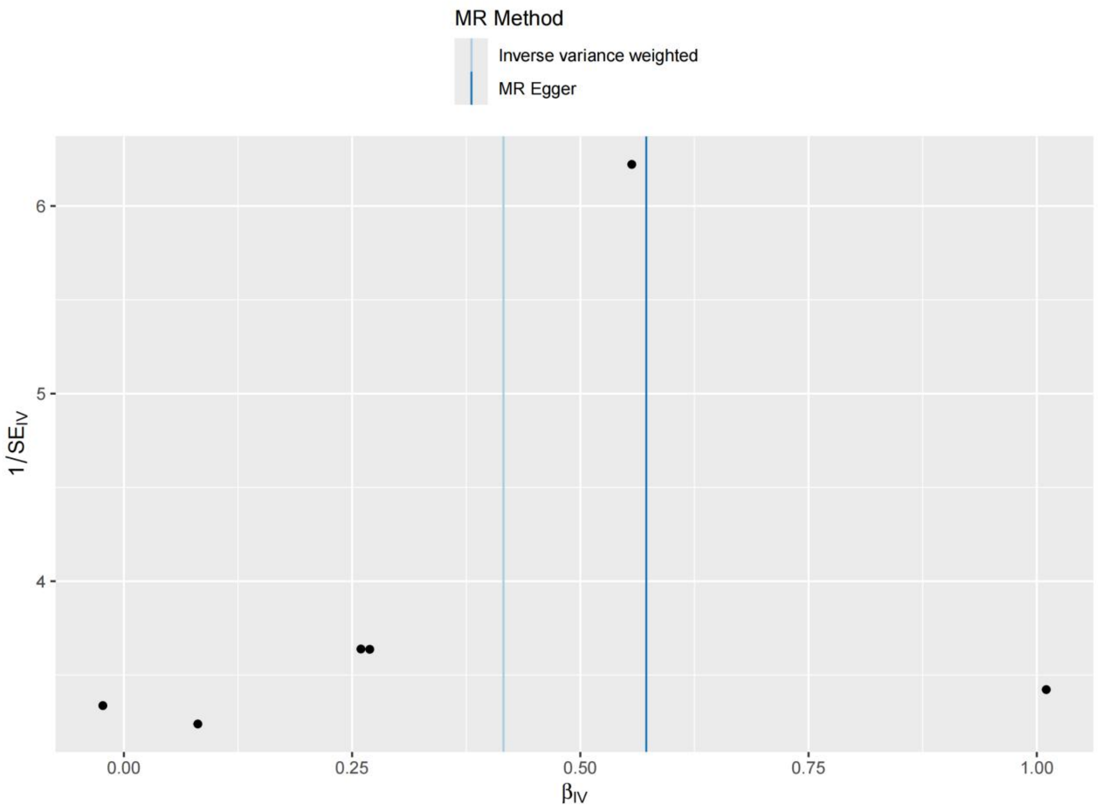

MR Method
Inverse variance weighted
MR Egger
6
5
4
1/SE$_{IV}$
0.00
0.25
0.50
0.75
1.00
β$_{IV}$

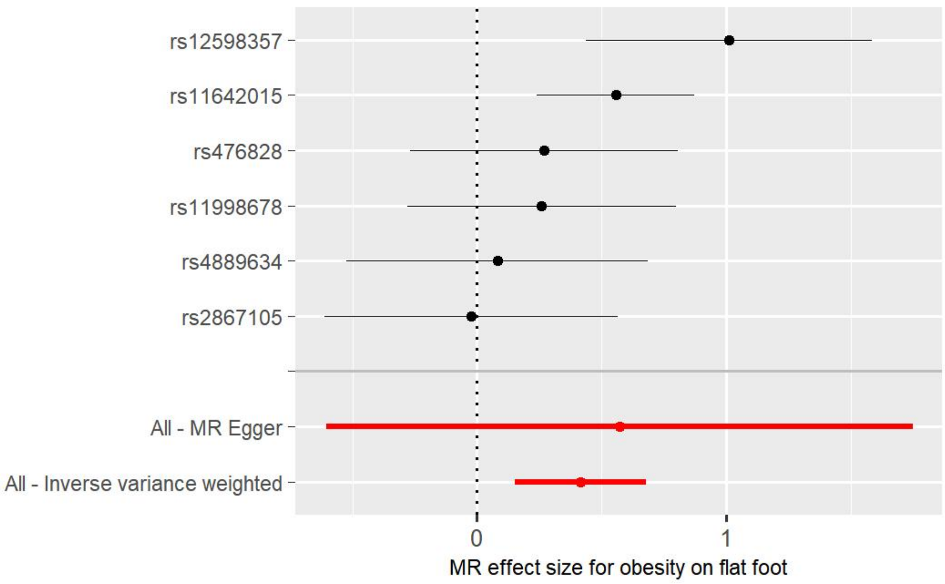

rs12598357
rs11642015
rs476828
rs11998678
rs4889634
rs2867105
All - MR Egger
All - Inverse variance weighted
0
1
MR effect size for obesity on flat foot

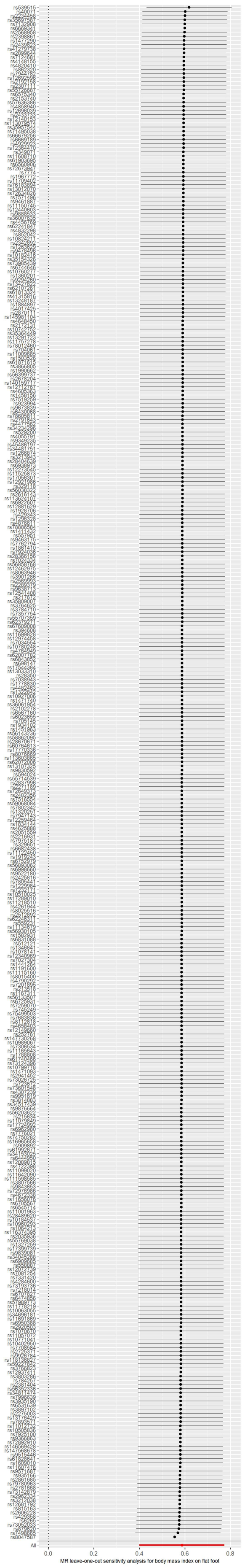

All
0.0
0.2
0.4
0.6
0.8
MR leave-one-out sensitivity analysis for body mass index on flat foot

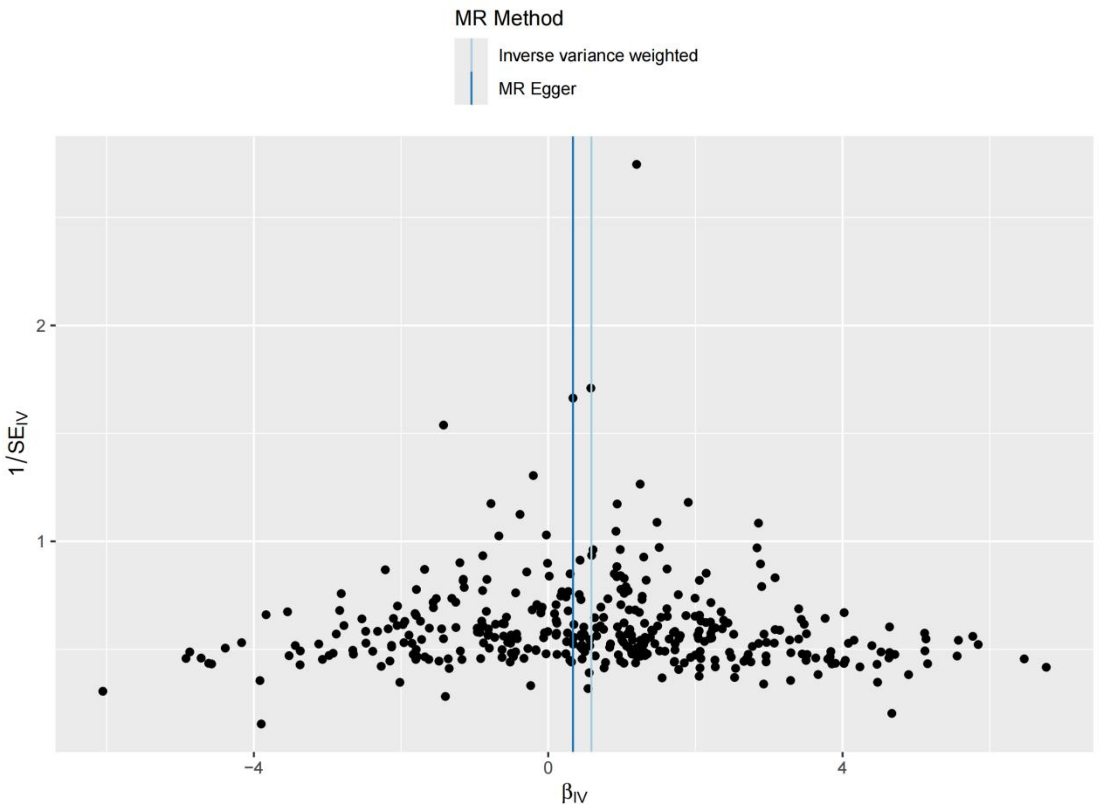

MR Method
Inverse variance weighted
MR Egger
2
1
$1/SE_{IV}$
−4
0
4
β_{IV}

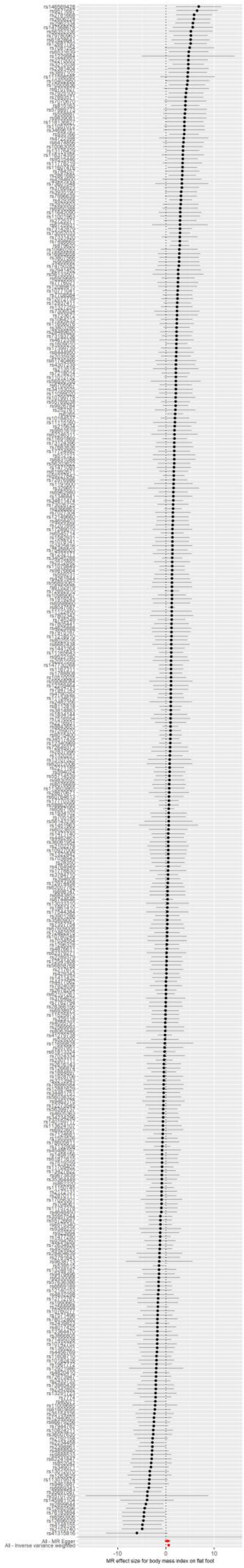
All - MR Egger
All - Inverse variance weighted
-10
0
10
MR effect size for body mass index on flat foot

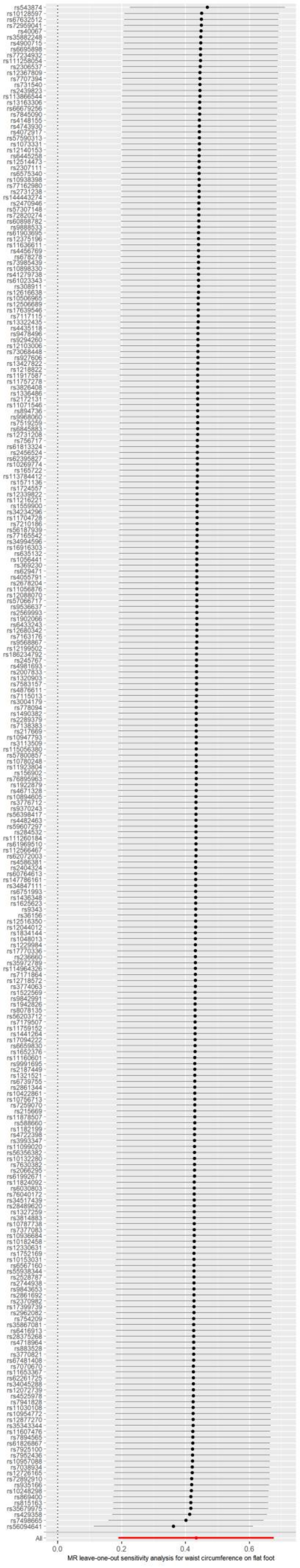
All
0.0
0.2
0.4
0.6
MR leave-one-out sensitivity analysis for waist circumference on flat foot

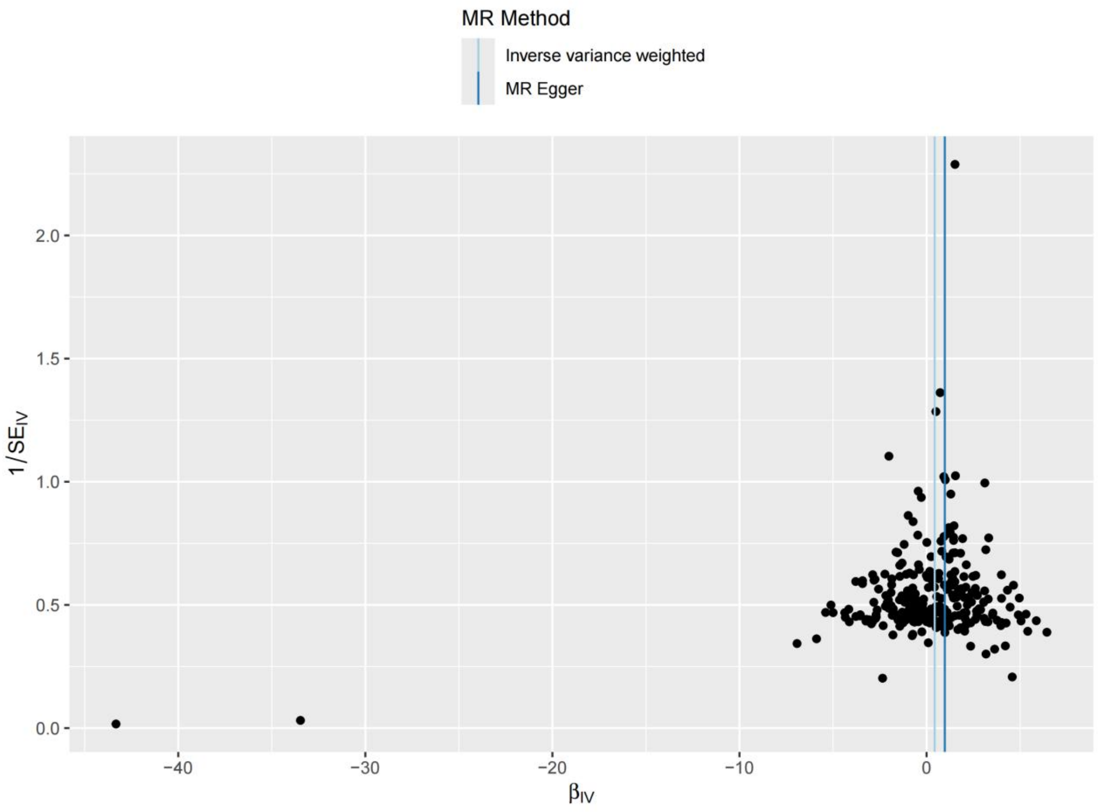
MR Method
Inverse variance weighted
MR Egger
1/SE_IV
β_IV
2.0
1.5
1.0
0.5
0.0
−40
−30
−20
−10
0

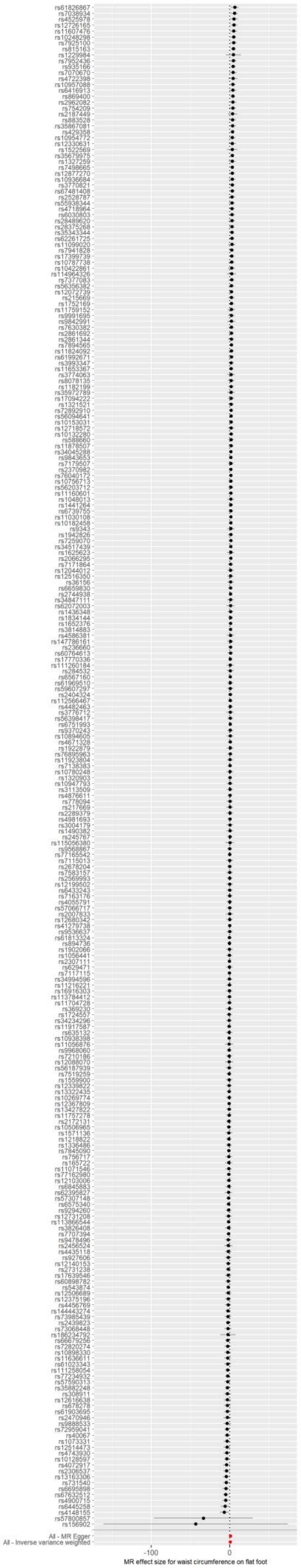

All - MR Egger
All - Inverse variance weighted
-100
0
MR effect size for waist circumference on flat foot

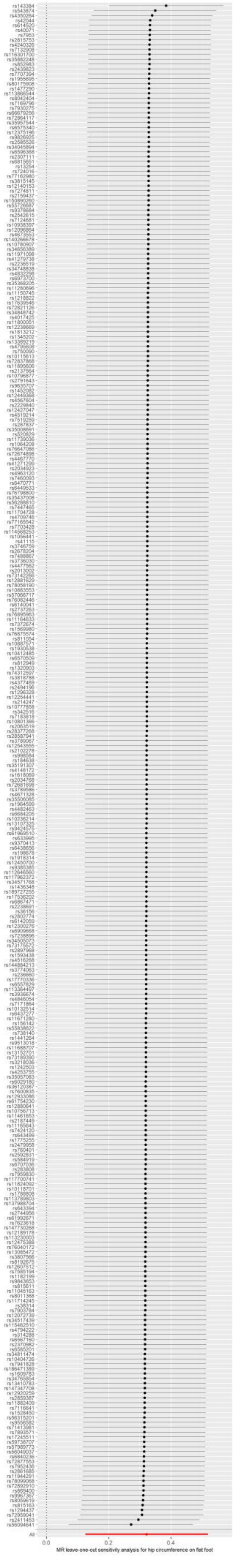
All
0.0
0.2
0.4
MR leave-one-out sensitivity analysis for hip circumference on flat foot

foot.

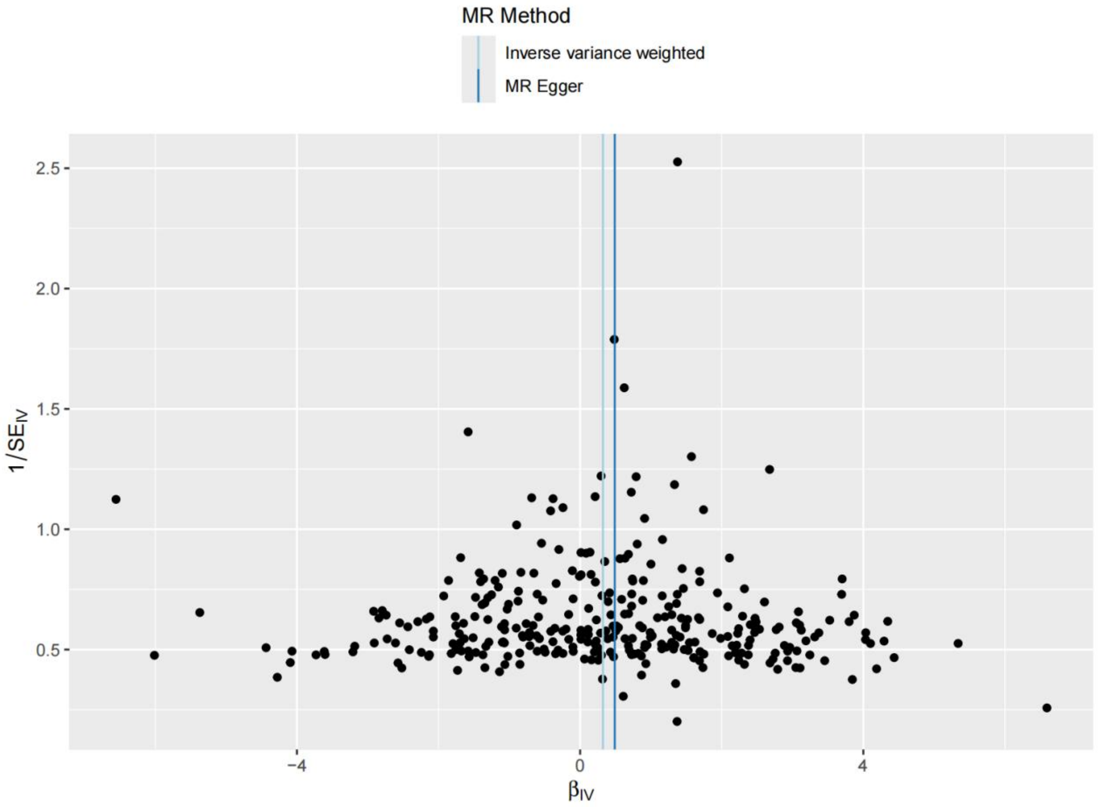

MR Method
Inverse variance weighted
MR Egger
2.5
2.0
1.5
1.0
0.5
1/SE_IV
−4
0
4
β_IV

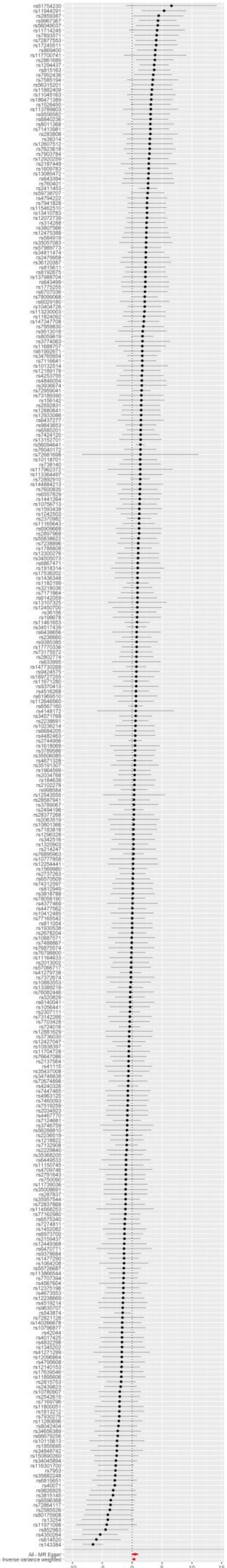
All - MR Egger
All - Inverse variance weighted
-10
-5
0
5
10
15
MR effect size for hip circumference on flat foot

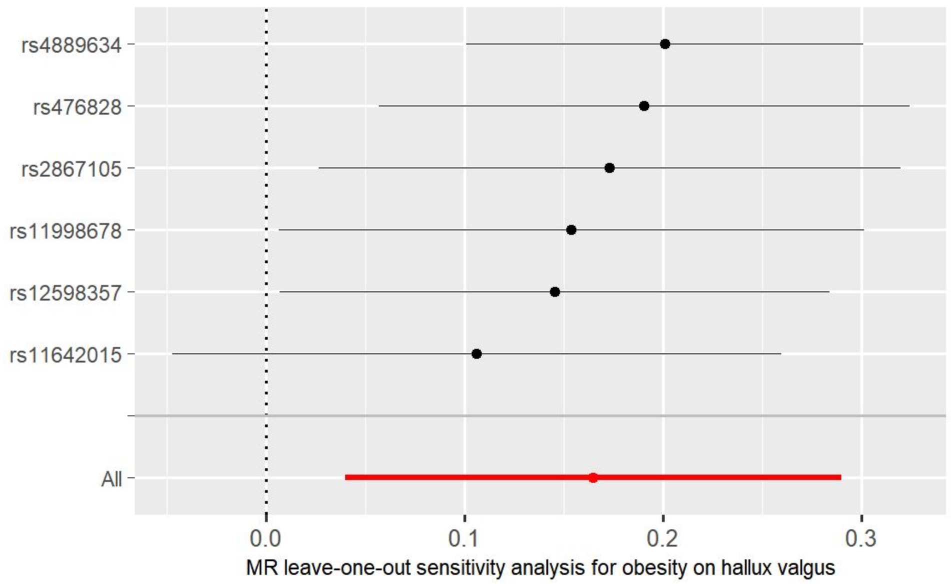
rs4889634
rs476828
rs2867105
rs11998678
rs12598357
rs11642015
All
0.0
0.1
0.2
0.3
MR leave-one-out sensitivity analysis for obesity on hallux valgus

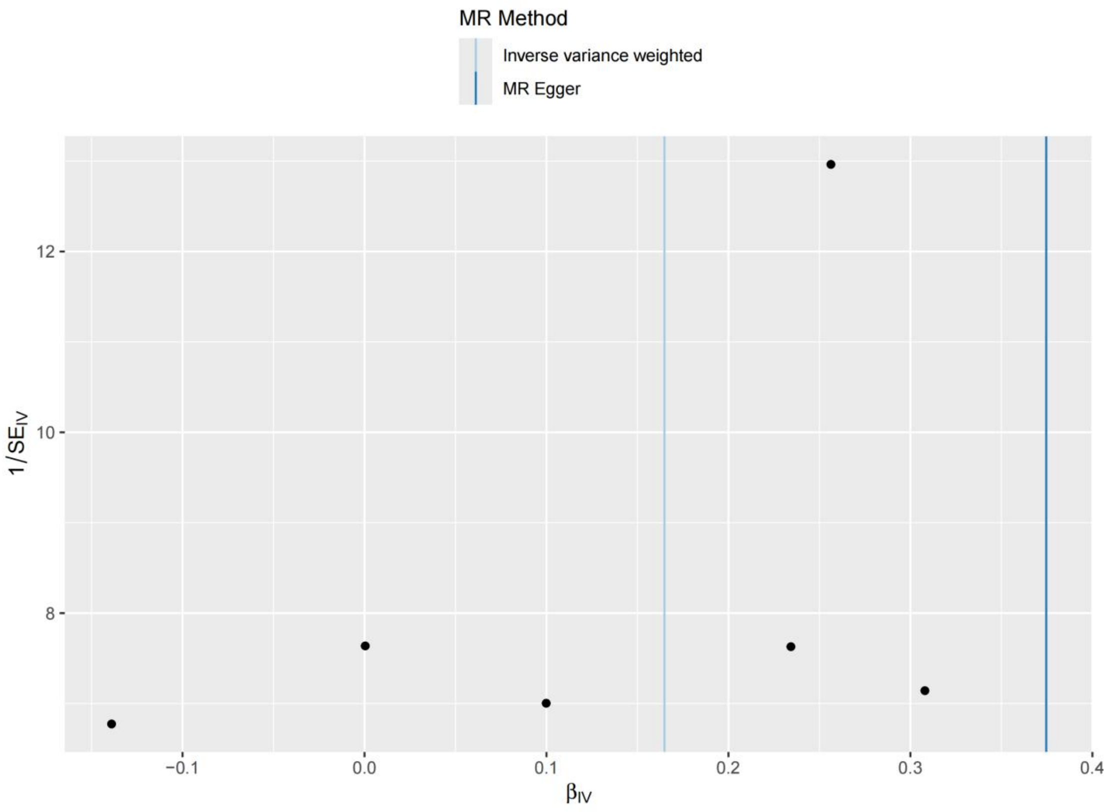

MR Method
Inverse variance weighted
MR Egger
1/SE_IV
12
10
8
−0.1
0.0
0.1
0.2
0.3
0.4
β_IV

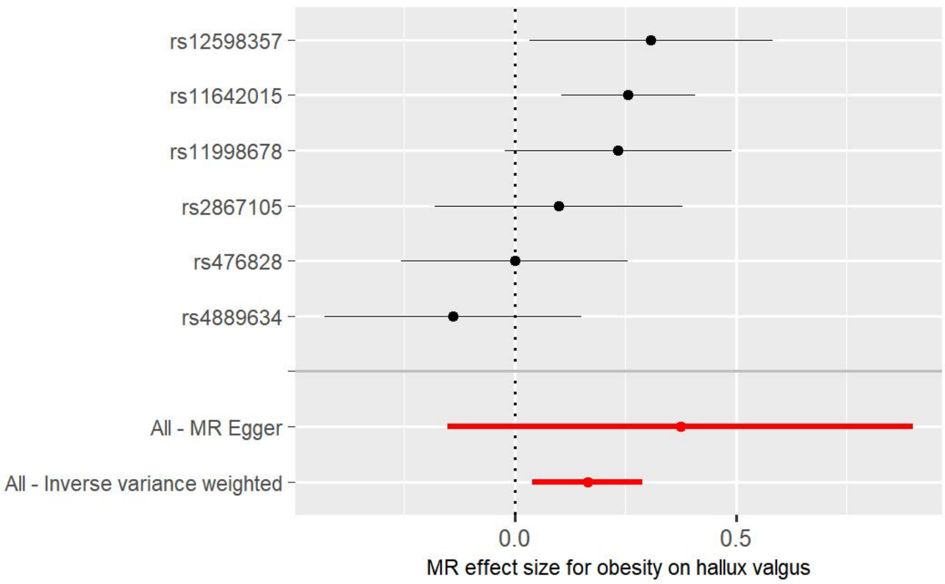
rs12598357
rs11642015
rs11998678
rs2867105
rs476828
rs4889634
All - MR Egger
All - Inverse variance weighted
0.0
0.5
MR effect size for obesity on hallux valgus

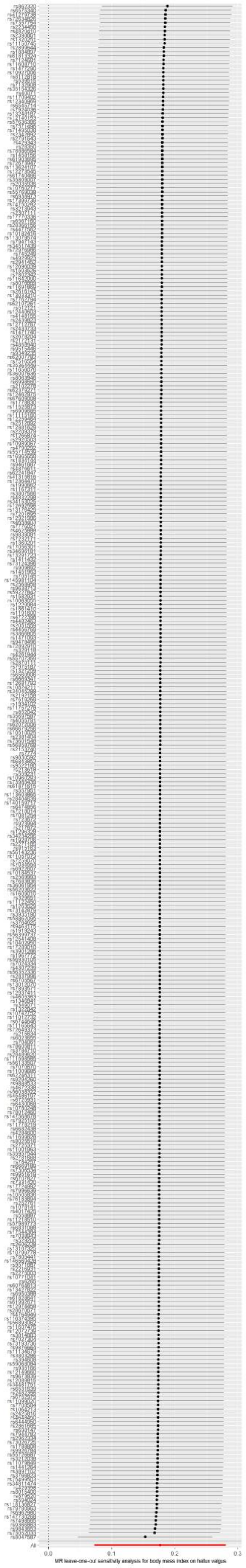
All
0.0
0.1
0.2
0.3
MR leave-one-out sensitivity analysis for body mass index on hallux valgus

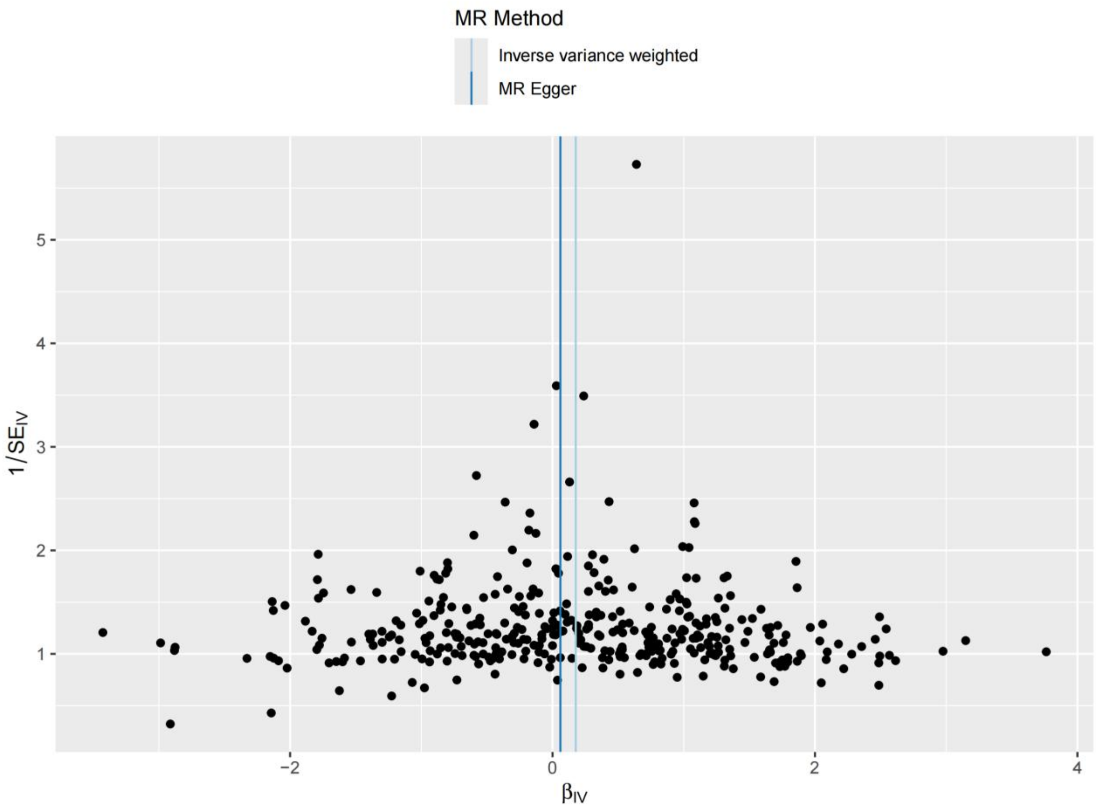

MR Method
Inverse variance weighted
MR Egger
5
4
3
2
1
1/SE_IV
−2
0
2
4
β_IV

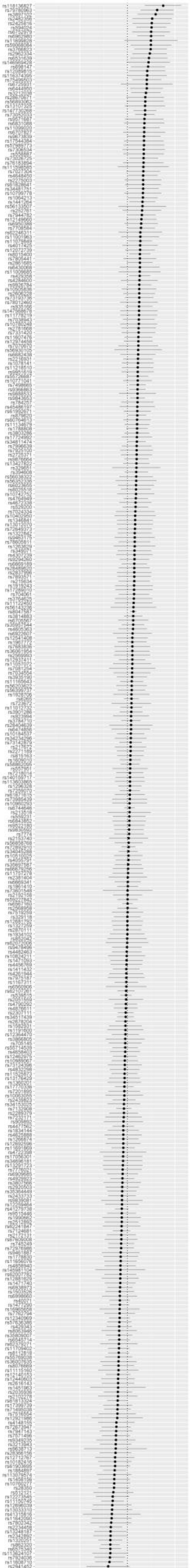

All - MR Egger
All - Inverse variance weighted
-5
0
5
MR effect size for body mass index on hallux valgus

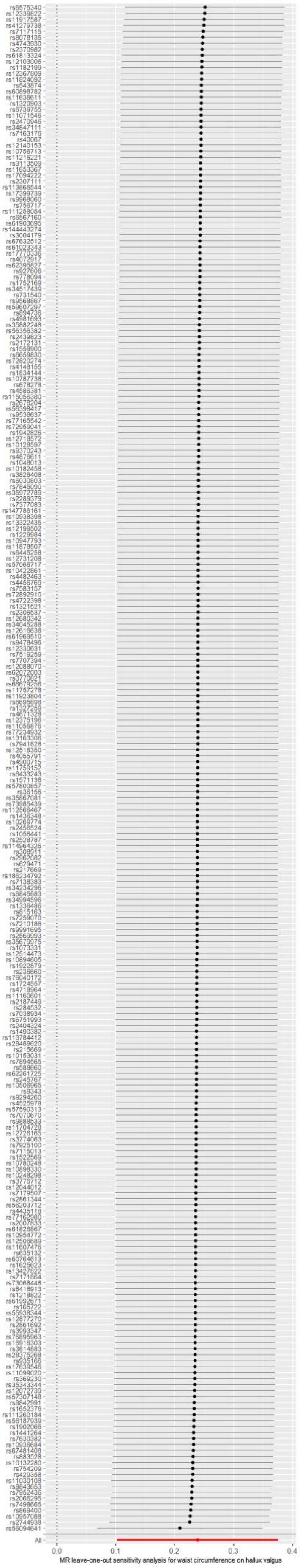
All
0.0
0.1
0.2
0.3
0.4
MR leave-one-out sensitivity analysis for waist circumference on hallux valgus

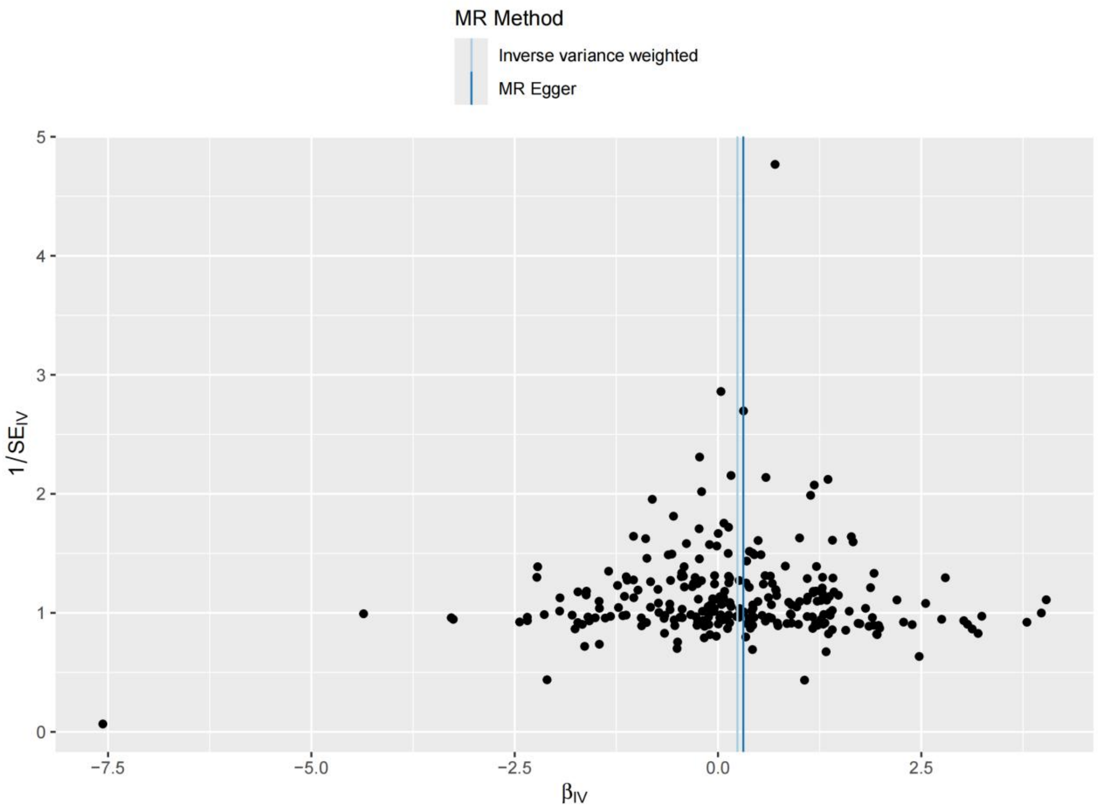
MR Method
Inverse variance weighted
MR Egger
5
4
3
2
1
0
1/SE$_{IV}$
−7.5
−5.0
−2.5
0.0
2.5
β$_{IV}$

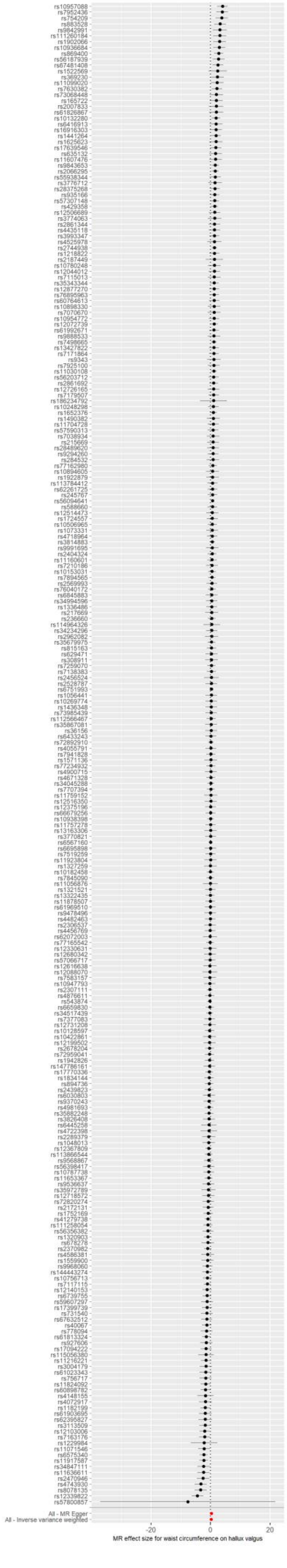

All - MR Egger
All - Inverse variance weighted
-20
0
20
MR effect size for waist circumference on hallux valgus

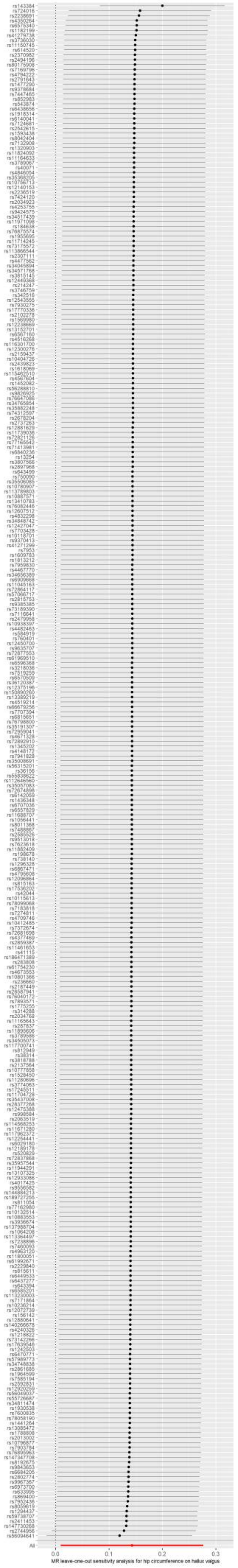
All
0.0
0.1
0.2
0.3
MR leave-one-out sensitivity analysis for hip circumference on hallux valgus

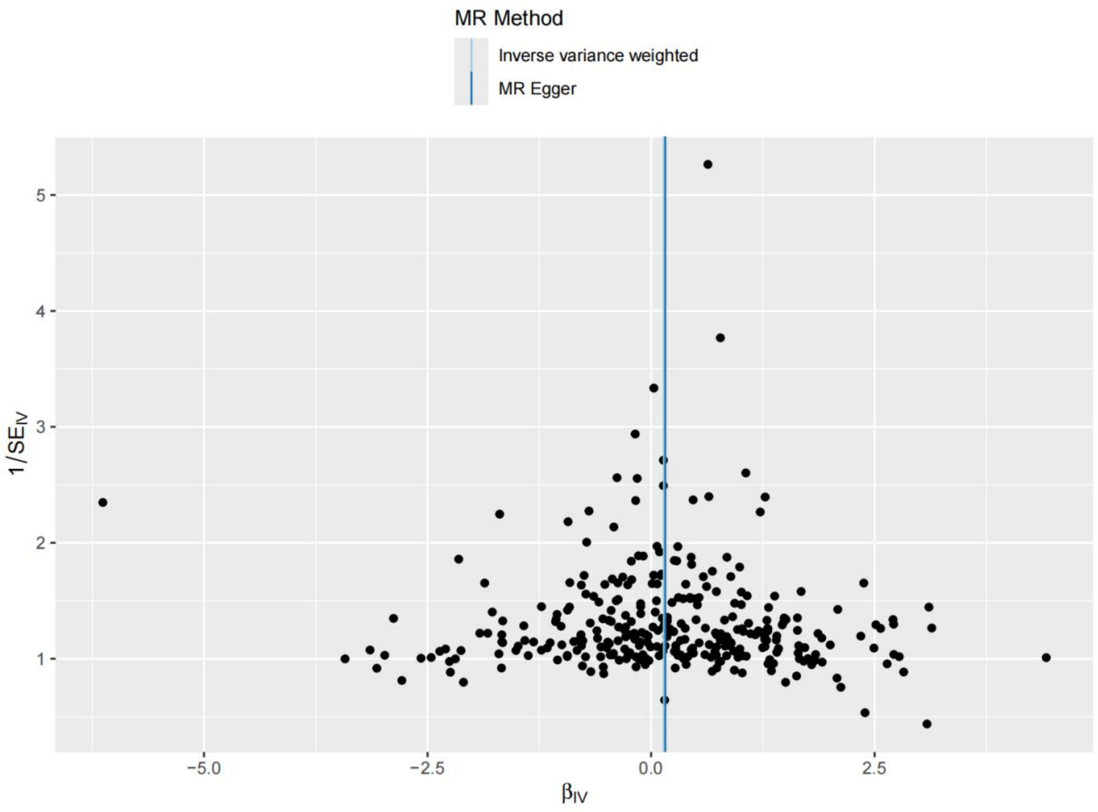
MR Method
Inverse variance weighted
MR Egger
5
4
3
2
1
$1/SE_{IV}$
−5.0
−2.5
0.0
2.5
β_{IV}

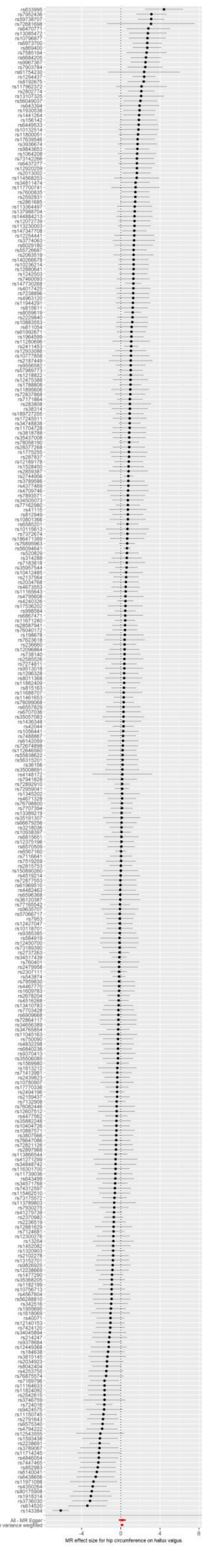

All - MR Egger
All - Inverse variance weighted
-4
0
4
8
MR effect size for hip circumference on hallux valgus
